# Training Load, Aerobic Capacity and Their Relationship With Wellness Status in Recreational Trail Runners

**DOI:** 10.3389/fphys.2019.01189

**Published:** 2019-09-13

**Authors:** Sérgio Matos, Filipe Manuel Clemente, António Brandão, Joel Pereira, Thomas Rosemann, Pantelis Theodoros Nikolaidis, Beat Knechtle

**Affiliations:** ^1^School of Sport and Leisure, Polytechnic Institute of Viana do Castelo, Melgaço, Portugal; ^2^Unidade de Investigação e Treino em Trabalhos em Alturas e Atividades de Ar Livre, Melgaço, Portugal; ^3^Instituto de Telecomunicações, Delegação da Covilhã, Covilhã, Portugal; ^4^Institute of Primary Care, University of Zurich, Zurich, Switzerland; ^5^Exercise Physiology Laboratory, Nikaia, Greece; ^6^Medbase St. Gallen Am Vadianplatz, St. Gallen, Switzerland

**Keywords:** training monitoring, session-rated of perceived exertion, global positioning system, performance, sports training

## Abstract

The present study aimed to analyze the relationship between variables related to the internal and external loads of training and competition races as well as to athletes’ perceptions of well-being measured throughout the course of a 4-week mesocycle. It also aimed to analyze the intra- and inter-week variations in terms of training load and well-being. The study included the participation of 47 male recreational athletes competing in the national championships of trail running in Portugal (age: 34.85 ± 8.88 years; height: 1.77 ± 0.58 m; body mass: 65.89 ± 3.17 kg). During the 4 weeks, subjective perception of effort (RPE), training time (min), session-RPE (sRPE), distance covered (km), and perception of well-being (Hooper’s questionnaire) were monitored. Weekly RPE was greater in week 1 than in week 3 (*p* = 0.001; *d* = 0.563, small effect). Moreover, weekly sRPE was greater in week 1 than in week 2 (*p* = 0.001; *d* = 0.441, small effect). The correlations between the well-being variables and RPE that were found to be significant with small magnitudes are those between sleep and RPE (*r* = 0.287; *p* = 0.001), stress and RPE (*r* = 0.217; *p* = 0.001), fatigue and RPE (*r* = 0.191; *p* = 0.001), muscle soreness and RPE (*r* = 0.240; *p* = 0.001), and Hooper’s index and RPE (*r* = 0.279; *p* = 0.001). Among the variables of the Cooper test and the competition race load, it was verified that VO_2max_ had a negative correlation of an average magnitude with pace (*r* = −0.396, *p* = 0.015). The findings of the study suggest that small variations in training stimulus during the period of analysis and increases in maximal oxygen uptake result in improvements in the performance of trail running athletes when considering the running speed in the race.

## Introduction

Changes in training load – particularly in the frequency, duration, and intensity of training sessions – are associated with the principle of training stimulus variability that seeks to optimize sports performance (Halson, 2014a). Training load monitoring can be categorized into two forms: external load and internal load (Malone et al., 2015). External load is understood as the physical repercussions of training performed by an athlete, encompassing indicators such as distance, duration, and race intensity (Impellizzeri et al., 2005). Internal load is associated with the biological response of the athlete to the external load imposed by training (Bourdon et al., 2017).

In the training’s prescription, it is essential that the external and internal loads be appropriate and that there is a balance between them, allowing for improvements in the performance of the athlete and for the reduction of overload or underload (Bartlett et al., 2017). The correct planning of the training load through microcycles allows an approximation of the training regarding the requirements of races (Phibbs et al., 2018), causing fundamental specific adaptations in the athlete (Manzi et al., 2010).

The monitoring of training loads requires an accurate and reliable evaluation of the determinants of the training process (Roos et al., 2013). However, the use of different methods and/or techniques is dependent on the context, namely considering the applicability and the resources. As an example, the internal load can be more objectively measured by using heart rate sensors or collecting a blood sample to determine the blood lactate (Twist and Highton, 2013). However, such methods are somehow invasive or not practical in some contexts. On the other hand, subjective scales of intensity (e.g., rated of perceived exertion) have been presenting very good levels of validity and reliability, are less invasive and more practical in realistic training scenarios (Haddad et al., 2017). Similarly, the external load quantification is also dependent on the context and will provide different information than internal load, mainly considering specific sports that require a great perception of the pace and intensity of running. For these cases, the global positioning systems are often used considering that may provide complementary information to coaches and athletes (Halson, 2014a).

Despite the unquestionable importance of quantification of the load to regulate the training process, the monitoring cycle of athletes does not finish with a simple quantification of the load. Other parameters related with the impact of the training stimulus on athletes are also a part of the monitoring process, namely considering the well-being parameters that include, among others, the perception of delayed onset muscle soreness (DOMS), fatigue, stress, or sleep quality (Hooper and Mackinnon, 1995). In this sense, the literature refers to well-being questionnaires as a good indicator of the evaluation of these variables, and the Hooper questionnaire (Hooper and Mackinnon, 1995) as being pointed out as a good tool to estimate the impact and to manage the dose of training in athletes.

Despite a great number of publications considering the training load quantification and well-being determination, the great majority of the studies are related to team sports (Roos et al., 2013; Malone et al., 2017) while just a few, to the best of our knowledge, are dedicated to individual sports (Stellingwerff, 2012; Hernández-Cruz et al., 2017). Among individual sports, the trail running practice has been increasing in the last few years and is a sport with an apparent necessity of load management considering the great distances covered by the athletes. This sport can be characterized as a mountain run (Saugy et al., 2013) with race distances that may vary according to the type of competition, ranging between 10 and 894 km (Rowlands et al., 2012). Trail running races are competitions that can last for several hours or even days because of accumulated unevenness and terrain specificity, with times varying from athlete to athlete (Easthope et al., 2014).

Due to the specificity of trail running, researches have been carried out to characterize the load and the physiological requirements derived from races (Vernillo et al., 2016). Usually, maximal oxygen uptake between 60 and 85 ml·kg^−1^·min^−1^ can be found in this type of athletes (Gordon et al., 2017). Therefore, it seems reasonable to assume that the training process should be adjusted to the requirements of the race and must be properly varied during the weeks aiming to fit the load with the performance expectations of the athletes. Despite this necessity, there is a lack of evidence about how athletes manage and apply the load. This is particularly important because a great number of these athletes are non-professional (recreational) and for that reason, it is important to characterize how they manage the load during the training and identify the variations of well-being parameters during the week.

To the best of our knowledge, no information has been reported about the intra- and inter-week variations of training load and well-being of trail runners. Based on that, the first purpose of our study was to characterize the training load (internal and external) and well-being parameters of trail runner athletes during a mid-season and competitive mesocycle of 4 weeks. As the second purpose of this study, we tested possible relationships (correlations) between aerobic capacity of athletes (estimated by a field-based test), performance in races (pace), and the training load variables, aiming to determine if the training process (namely intensity and volume) can be associated with the aerobic capacity and performance of these recreational athletes.

## Materials and Methods

### Participants

Forty-seven Portuguese male trail running athletes (average age = 34.85 ± 8.88 years; height: 177.34 ± 5.81 cm; weight: 65.89 ± 3.17 kg; experience: 4.72 ± 2.11 years) participated in this study. On average, the athletes covered 35,159 m and trained for over 206 min per week. The absence of injuries during the study and the accomplishment of at least one and at most three races of the national championship of trail running were the criteria of inclusion. All participants provided informed consent in accordance with the recommendations of the Declaration of Helsinki for human study. The study was also approved by the local ethical committee (Polytechnic Institute of Viana do Castelo, School of Sport and Leisure) with the code number IPVC-ESDL171003.

### Design

The external and internal loads and the well-being of trail running athletes were monitored throughout the month of November 2017, during which 26 athletes participated in races of the national trail running championships. Despite participating in national trail running championships, the category of these athletes is recreational based on the fact that they are not professional and perform three sessions/week or less. However, for inclusion in this study, those reported less than three sessions per week were excluded from the analysis. From the athletes that participated in races, 65.4% competed in one race, 23.1% in two races, and 11.5% in three races. The races varied from a minimum of 10 km to a maximum of 300 km. The pace (min/km) made by the athletes during each race was collected to further correlations between aerobic capacity (as indicator of fitness level) and performance in race. In the week before the training load and well-being monitoring started, the 12-min Cooper test was implemented. During the training mesocycle, athletes were required to fill out the Hooper questionnaire before training sessions and races and to fill out the Borg scale after the end of training sessions and races. Both questionnaires were completed using an online form. External load was monitored using GPS (Global Positioning System) devices.

### Data Collection: Global Positioning System

During the training sessions, the athletes used watches with GPS technology, enabling the collection of information regarding horizontal movement. The Polar V800 (37 mm × 56 mm × 12.7 mm and weight: 79 g) (Roos et al., 2017) was used based on its validity for the collection of positional information.

### Hooper Index

The Hooper index (HI) questionnaire for assessing athletes’ well-being was administered individually, 30 min before training sessions and races, for the variables of sleep quality, stress, fatigue, and muscle soreness. Answers were given using scales of 1–7. For the variables of fatigue, stress, and muscle soreness, 1 = very, very low, and 7 = very, very high. For sleep quality, 1 = very, very bad and 7 = very, very good (Hooper and Mackinnon, 1995).

### Rated of Perceived Exertion

The rated of perceived exertion (RPE) quantified by using the CR-10 Borg’s scale (Borg, 1998) was used as a measure of exercise intensity. On the CR-10 Borg’s scale, the 1 = very, very easy and 10 = extremely hard. The CR-10 Borg’s scale was firstly introduced to the participants aiming to familiarize them with the scale. After that, they have used the scale for 2 weeks without including the data in the study just aiming to increase the familiarization and the accuracy of the athlete’s answers.

After such period, and during the data collection, the athletes scored the RPE 30 min after the end of training session in a dedicated online form built for the effect. Moreover, they reported the time of the session in minutes. Using both information (i.e., RPE score and time of the session), it was possible to determine the session-RPE (sRPE) that represents the overall internal load of the session by multiplying the RPE score for the time of the session in minutes (Foster et al., 2001). The sRPE has been used as a valid and reliable measure of internal load (Haddad et al., 2017). The data were collected in all training sessions that occurred in the period of data collection, thus the sRPE was calculated on a daily basis.

### The 12-min Cooper Test

A Cooper test with a duration of 12 min was performed for the estimation of cardiorespiratory capacity and the maximal oxygen uptake (VO_2max_). The test was performed while athletes were running or walking without interruption, and the total distance covered in the 12 min was recorded. All the participants reported previous experience and familiarization in this specific test, considering their previous participation in performance assessments.

A 5-min warm-up run was performed with a 10-min interval between the warm-up run and the test. All athletes performed the test in the same place between 10:00 a.m. and noon, with no precipitation at a temperature of 14°C and with a relative humidity of 45%. The test took place 72 h after the previous race or training session. The test was performed in an official athletic track and the distance covered by each athlete was collected immediately after the 12-min Cooper test. The distance covered (m) was one of the measures associated with the performance in the Cooper test. Moreover, using the distance on the Cooper test and the equation proposed in Bandyopadhyay (Bandyopadhyay, 2014), the maximal oxygen uptake of the athletes was estimated.

### Data Analyses and Statistics

**Figure 1:**
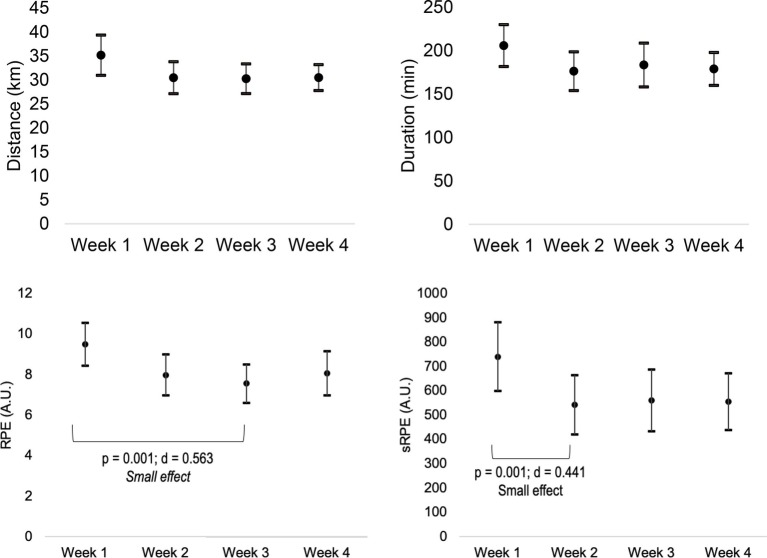
Weekly variations of accumulated (sum of the A.U. of all sessions of each week) RPE and session-RPE (sRPE).

**Figure 2:**
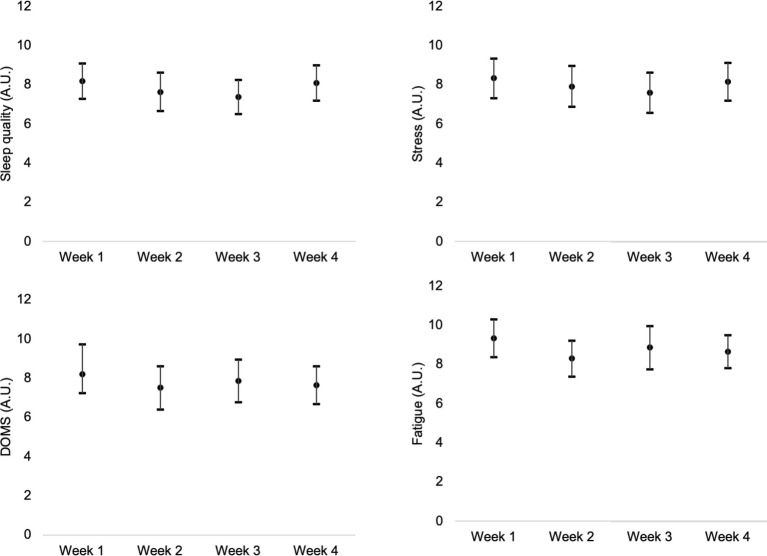
Weekly variations of accumulated (sum of the scores of all sessions of each week) stress, fatigue, DOMS, and sleep quality.

**Figure 3:**
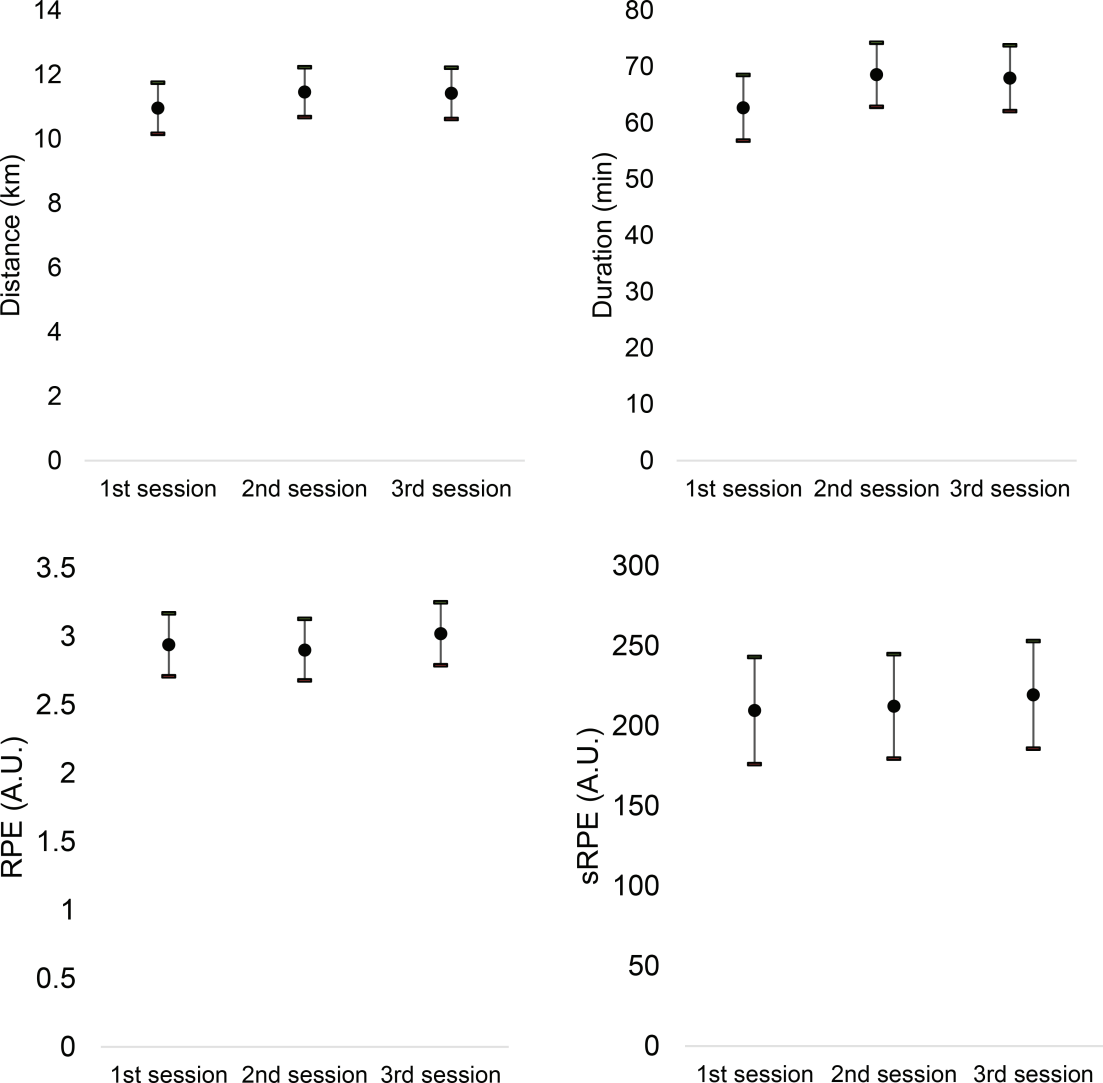
Intra-week variations of distance, duration, RPE, and session-RPE (sRPE) (averages of 1st, 2nd, and 3rd training sessions of the week).

**Figure 4:**
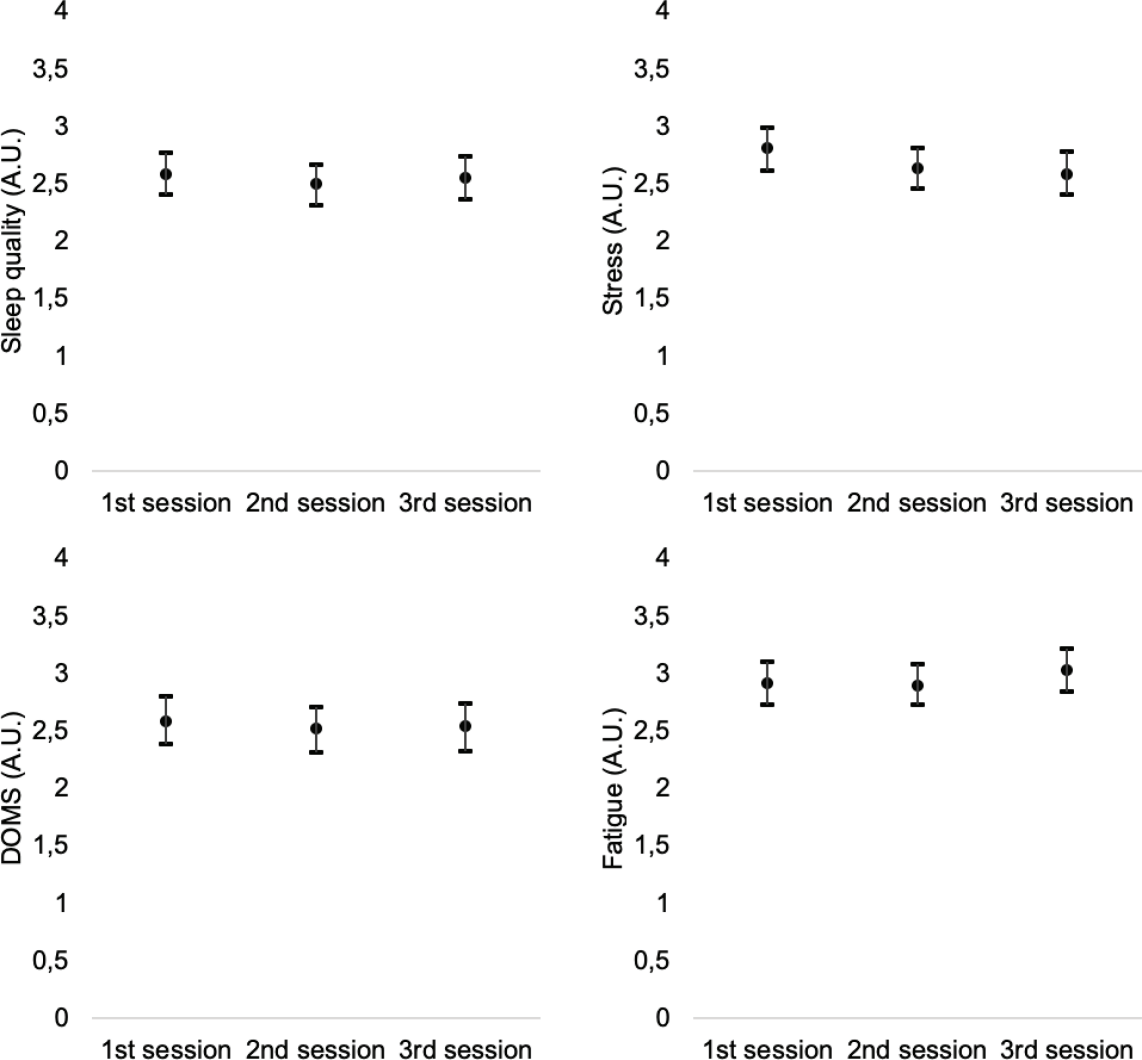
Intra-week variations of sleep quality, stress, fatigue, and DOMS (averages of 1st, 2nd, and 3rd training sessions of the week).

Descriptive statistics were presented in the form of mean, standard deviation, and 95% confidence intervals (presented in the Figures 1–4). The weekly RPE and well-being variables were treated as the average of the RPE and well-being variables in each week for each athlete and then integrated into the mean of the participants (Figures 3, 4). The weekly accumulated load and well-being [sum of the arbitrary units (A.U.) of all sessions of each week] were also calculated for each athlete and then integrated into the mean of the participants (Figures 1, 2).

Inter-week (comparisons of the weekly average of each measure between the 4 weeks) and intra-week (comparisons of the 4 weeks’ average of each measure in each training session) comparisons were tested with one-way repeated-measures ANOVA after confirmation of the assumptions of normality and homogeneity of the samples. The partial eta squared ($\eta^{2}$) tested the effect size of the repeated-measures ANOVA. The magnitude inferences of $\eta^{2}$ were defined as (Ferguson, 2009): no effect ($\eta^{2}$ < 0.04), small effect (0.05 < $\eta^{2}$ < 0.25), moderate effect (0.26 < $\eta^{2}$ < 0.64), or strong effect ($\eta^{2}$ > 0.65). The Tukey HSD *post hoc* test and the Cohen’s *d* tested the significances and the effect size of differences between factors. The following magnitude inferences were made for the Cohen’s *d*: 0.0–0.2, trivial effect; 0.2–0.6, small effect; 0.6–1.2, moderate effect; and 1.2–2.0, large effect.

For the study of associations between well-being variables, training load; aerobic capacity; and the race performance, Pearson’s *r* test was performed. In the particular case of correlations tested with the performance in the races, it was used a mean of the pace of each participant in the races in he has participated. According to the Hopkins classification followed for the study of the magnitude of correlations, correlation values were classified as follows (Hopkins et al., 2009): [0.0,0.1], trivial; (0.1,0.3], small; (0.3,0.5], moderate; (0.5,0.7], large; (0.7,0.9], very large; and (0.9,1.0], nearly perfect. Statistical procedures were performed in the statistical software SPSS (IBM, USA, version 23.0) for a significance level of 5%.

## Results

### Intra- and Inter-Week Variations of Training Load and Well-Being

The descriptive statistics of accumulated (sum of the sessions of each week) training load parameters and well-being variables can be found in Figures 1, 2, respectively.

Inter-week (changes between weeks) differences for the weekly average of RPE (*p* = 0.011; $\eta^{2}$ = 0.078, small effect) and sRPE (*p* = 0.025; $\eta^{2}$ = 0.065, small effect) were found. Weekly RPE was greater in week 1 than in week 3 (*p* = 0.001; *d* = 0.563, small effect). Moreover, weekly sRPE was greater in week 1 than in week 2 (*p* = 0.001; *d* = 0.441, small effect). No significant changes in weekly duration (*p* = 0.12; $\eta^{2}$ = 0.40, no effect) and distance (*p* = 0.062; $\eta^{2}$ = 0.062, small effect) were found.

No significant changes were found in weekly sleep (*p* = 0.389; $\eta^{2}$ = 0.030, no effect), weekly stress (*p* = 0.537; $\eta^{2}$ = 0.022, no effect), weekly fatigue (*p* = 0.319; $\eta^{2}$ = 0.035, no effect), and weekly muscle soreness (*p* = 0.562; $\eta^{2}$ = 0.020, no effect).

Intra-week (changes within week) analysis of training load and well-being parameters can be found in Figures 3, 4, respectively. Repeated-measures ANOVA did not reveal significant changes between the training sessions of the week in the distance (*p* = 0.618; $\eta^{2}$ = 0.002, no effect), duration (*p* = 0.303; $\eta^{2}$ = 0.005, no effect), RPE (*p* = 0.751; $\eta^{2}$ = 0.001, no effect), and internal load (*p* = 0.915; $\eta^{2}$ = 0.001, no effect).

Intra-week comparisons of well-being variables also revealed no significant changes between training session in sleep quality (*p* = 0.776; $\eta^{2}$ = 0.001, no effect); stress (*p* = 0.233; $\eta^{2}$ = 0.006, no effect); fatigue (*p* = 0.557; $\eta^{2}$ = 0.002, no effect); muscle soreness (*p* = 0.852; $\eta^{2}$ = 0.001, no effect); and Hooper index (*p* = 0.733; $\eta^{2}$ = 0.001, no effect).

### Association Between Well-Being and Performance in Training

The mean values of the RPE, s-RPE, and well-being variables during 4 weeks of training of trail running athletes can be seen in Table 1.

**Table 1:** Descriptive statistics (*M* ± SD) of well-being variables and training load during the mesocycle (average of the training session).

	Mean	SD
Sleep (A.U.)	2.55	1.15
Stress (A.U.)	2.68	1.16
Fatigue (A.U.)	2.95	1.16
DOMS (A.U.)	2.55	1.27
Hooper index (A.U.)	10.72	3.99
Distance (km)	11.28	5.31
Duration (min)	66.43	39.07
RPE (A.U.)	2.95	1.54
sRPE (A.U.)	213.79	223.95

*DOMS, delayed onset muscle soreness; RPE, rate of perceived exertion in the CR-10 Borg’s scale; sRPE, session-RPE representing the multiplication of RPE by the time in minutes; A.U., arbitrary units*.

The correlation between well-being variables and training load variables was identified in order to identify possible associations between these variables throughout the training process. The results of Pearson’ correlation coefficients *r* can be found in Table 2.

**Table 2:** Correlation values (*r*) between well-being variables and training load along the mesocycle.

	Distance (km)	Duration (min)	RPE (A.U.)	sRPE (U.A.)
Sleep (A.U.)	0.207^b^	0.153^b^	0.287^b^	0.249^b^
Stress (A.U.)	−0.007	−0.038	0.217^b^	0.079
Fatigue (A.U.)	0.068	0.012	0.191^b^	0.109^a^
DOMS (A.U.)	0.134^b^	0.098^a^	0.240^b^	0.193^b^
Hooper index (A.U.)	0.120^b^	0.068	0.279^b^	0.188^b^

*RPE, rated of perceived exertion in the CR-10 Borg’s scale; DOMS, delayed onset muscle soreness; sRPE, session-RPE representing the multiplication of RPE by the time in minutes*.

a *Significant correlation at p < 0.05*.

b *Significant correlation at p < 0.01*.

### Physical Variables and Performance in Evidence

Table 3 presents the mean values of the participants who underwent the 12-min Cooper test and the races performed during the mesocycle.

**Table 3:** Descriptive statistics (*M* ± SD) of the 12-min Cooper test and race values during the mesocycle.

	Mean	SD
Cooper test 12-min (m)	3168.97	287.57
VO_2max_ (ml/kg/min^−1^)	59.56	6.43
RPE (A.U.)	6.15	2.24
Pace (min/km)	7.38	2.04

*RPE, rated of perceived exertion in the CR-10 Borg’s scale*.

The Pearson’s correlation analysis was performed between the performance variables in the Cooper test and the sports performance measured in the race (Table 4). Positive and significant mean values were found between Cooper 12-min (m) and RPE (*r* = 0.380, *p* = 0.017), as well as negative values with moderate magnitude between Cooper 12 -min and the pace (*r* = −0.395, *p* = 0.016). Similar results were observed between estimated VO_2max_ and RPE (*r* = 0.379, *p* = 0.017) and the pace (*r* = −0.396, *p* = 0.015).

**Table 4:** Correlation values (*r*) between the performance variables in the 12-min Cooper test and load during the races.

	RPE (A.U.)	Pace (min/km)
Cooper test 12-min (m)	0.380^a^	−0.395^a^
VO_2max_ (ml/kg/min^−1^)	0.379^a^	−0.396^a^

*RPE, rated of perceived exertion in the CR-10 Borg’s scale*.

a *Significant correlation at p < 0.05*.

## Discussion

Significant changes of RPE and s-RPE were found between the first and the third weeks and the first and the second weeks, respectively. Despite that, no more significant changes were found between weeks, possibly suggesting that there is a lack of progression in the stimulus and variability inter-week that is crucial to optimize the performance and to reduce the exposure to injuries (Gabbett, 2016). In fact a stabilization of the load may contribute to a performance plateau and, for that reason, it is interesting to identify that these athletes are not promoting (in a significative scale) the principles of variability and progression of the load based on the general absence of changes in the training load during the week and even the general comparisons between accumulated load over the weeks analyzed. One possible cause to observe such tendency can be the fact that during the period the athletes participated in races, and this may be constrained the variability within the mesocycle. As previously mentioned, the mesocycles occurred in mid-season during the competitive period; however, and depending on the goal of each athlete, some variability in terms of the training process may occur. This should be considered a limitation of the present study. Despite that, the descriptive statistics of the internal load revealed that the sRPE per training day was relatively similar to that of the previous reports in race athletes (Da Silva et al., 2014). Another evidence was that no intra-week changes (differences between sessions within the week) were found in training load or in well-being variables, suggesting that the training within the week is almost the same between training sessions, again revealing a lack of variability in the training stimulus.

The analysis of well-being variables and training load (Table 1) allowed us to observe possible associations between these two factors during the mesocycle. By analyzing Table 2 for correlation values, it can be seen that sleep quality has a significant small magnitude with all training load variables, indicating that sleep may have consequences related to athlete performance (Halson, 2014b). The stress variable only correlates in a significant way with RPE, although with a small magnitude, which may lead to an incorrect perception of the internal load resulting from the training sessions or races, and this may provide mismatched feedback for training monitoring (Halson, 2014a). Fatigue correlates significantly at a small magnitude with RPE and internal load variables, showing that high levels of RPE indicate the presence of fatigue (Gescheit et al., 2015). DOMS had a small magnitude with distance, RPE, and internal load. This correlation suggests that an athlete’s perception of muscle soreness is related to the impact of the race (Govus et al., 2017). The Hooper index scores correlate significantly, albeit with a small magnitude, with the variables of distance, RPE, and internal load. This result allows us to affirm that athletes who train or race with high Hooper index values are likely to have low levels of well-being, resulting in a reduction in performance (Gescheit et al., 2015).

Aerobic capacity of the athletes was tested to further correlations with performance in race. Our results in the 12-min Cooper test presented mean values of 3168.97 m. The values were similar with those reported by Kumar (2015). Also, mean values of 59.56 ± 6.43 ml·kg^–1^·min^–1^ (VO_2max_) were estimated in our recreational athletes. The correlation between the Cooper test and RPE showed positive and significant values, suggesting that a greater performance in Cooper may allow achieving higher intensities in training sessions. However, more interestingly, negative correlations were found between distance covered at Cooper test and estimated VO_2max_ with the pace in races, suggesting that greater aerobic capacity increases the intensity of running during official races. These results are in agreement with the literature regarding VO_2max_ as being the variable with the greatest effect on success in medium- and long-distance races (Bassett and Howley, 2000), being determinant to be succeeded.

The values obtained in the present recreational trail runners during races revealed a mean of perceived intensity of 6.15 ± 2.24 on the Borg scale. The pace of the athletes during races presented a mean of 7.38 ± 2.04 min/km. According to a study on running athletes by Dantas and Doria (2015), the pace was 4.05 min/km over a distance of 10 km, 4.21 min/km over the distance of a half marathon, and 4.48 min/km over the distance of a marathon. The differences between both values can be associated with the typology of long-running activities considering that trail running means to run in mountains with great variations in terms of terrain and accumulated unevenness involved.

Despite its contributions, our study had some limitations. For future studies, we recommend that heart rate during training sessions and competitions can be considered. Caloric intake should also be considered in order to determine the influence it has on the performance level. Moreover, hydration levels resulting from the excess body temperature of the athletes during a race should be also controlled. This can be also associated with the internal load in race considering that dehydration in trail running athletes causes increases in heart rate, which results in increases in fatigue levels and in an erroneous perception of effort. Finally, in the case of professional athletes, it would be important to compute some robust parameters associated with training load analysis, namely, the acute: chronic workload ratio, training monotony, and training strain.

This competitive 1-month analysis of trail running athletes demonstrated that well-being variables had small correlations between RPE and sRPE. Moreover, a negative correlation was observed between aerobic capacity measured in the Cooper test and the estimated VO_2max_ with the pace in race, demonstrating that increases in maximal oxygen consumption translate into improvements in the pace and performance of athletes.

## Conclusions

It was found that, generally, there are no significant changes of training load and well-being parameters within and between weeks. Small correlations were found between training load parameters and well-being variables. A third evidence was that moderate correlations between aerobic capacity and performance in race revealed that higher levels in maximum oxygen consumption (VO_2max_) reflect a decrease in pace (min/km) and, consequently, in performance improvements during races.

## Data Availability

The datasets for this manuscript are not publicly available because upon request from the first author. Requests to access the datasets should be directed to Sérgio Matos: sfcmatos@gmail.com.

## Ethics Statement

The study was also approved by the local ethical committee (Polytechnic Institute of Viana do Castelo, School of Sport and Leisure) with the code number IPVC-ESDL171003.

## Author Contributions

SM and FC conceived the study. FC and AB designed the study. SM collected data. FC analyzed and interpreted the data. SM, FC, and AB drafted the manuscript. SM, FC, AB, JP, PN, TR, and BK revised the manuscript and approved the final version.

### Conflict of Interest Statement

The authors declare that the research was conducted in the absence of any commercial or financial relationships that could be construed as a potential conflict of interest.

## References

[ref1] Bandyopadhyay A. (2014). Validity of Cooper’s 12-minute run test for estimation of maximum oxygen uptake in male university students. Biol. Sport 32, 59–63. 10.5604/20831862.1127283 25729151 PMC4314605

[ref2] Bartlett J. D. O’Connor F. Pitchford N. Torres-Ronda L. Robertson S. J. (2017). Relationships between internal and external training load in team-sport athletes: evidence for an individualized approach. Int. J. Sports Physiol. Perform. 12, 230–234. 10.1123/ijspp.2015-0791, PMID: 27194668

[ref3] Bassett D. R. Howley E. T. (2000). Limiting factors for maximum oxygen uptake and determinants of endurance performance. Med. Sci. Sports Exerc. 32, 70–84. 10.1097/00005768-200001000-00012, PMID: 10647532

[ref4] Borg G. (1998). Perceived exertion and pain scales. Champaign IL, USA: Human Kinetics.

[ref5] Bourdon P. C. Cardinale M. Murray A. Gastin P. Kellmann M. Varley M. C. (2017). Monitoring athlete training loads: consensus statement. Int. J. Sports Physiol. Perform. 12, S2-161–S2-170. 10.1123/IJSPP.2017-0208 28463642

[ref6] Da Silva D. F. Verri S. M. Nakamura F. Y. Machado F. A. (2014). Longitudinal changes in cardiac autonomic function and aerobic fitness indices in endurance runners: a case study with a high-level team. Eur. J. Sport Sci. 14, 443–451. 10.1080/17461391.2013.832802 23998661

[ref7] Dantas J. L. Doria C. (2015). Detection of the lactate threshold in runners: what is the ideal speed to start an incremental test? J. Hum. Kinet. 45, 217–224. 10.1515/hukin-2015-0022, PMID: 25964824 PMC4415835

[ref8] Easthope C. S. Nosaka K. Caillaud C. Vercruyssen F. Louis J. Brisswalter J. (2014). Reproducibility of performance and fatigue in trail running. J. Sci. Med. Sport 17, 207–211. 10.1016/j.jsams.2013.03.009 23660298

[ref9] Ferguson C. J. (2009). An effect size primer: a guide for clinicians and researchers. Prof. Psychol. Res. Pract. 40, 532–538. 10.1037/a0015808

[ref10] Foster C. Florhaug J. A. Franklin J. Gottschall L. Hrovatin L. A. Parker S. (2001). A new approach to monitoring exercise training. J. Strength Cond. Res. 15, 109–115. 10.1519/1533-4287(2001)015<0109:anatme>2.0.co;2 11708692

[ref11] Gabbett T. J. (2016). The training—injury prevention paradox: should athletes be training smarter and harder? Br. J. Sports Med. 50, 273–280. 10.1136/bjsports-2015-095788, PMID: 26758673 PMC4789704

[ref12] Gescheit D. T. Cormack S. J. Reid M. Duffield R. (2015). Consecutive days of prolonged tennis match play: performance, physical, and perceptual responses in trained players. Int. J. Sports Physiol. Perform. 10, 913–920. 10.1123/ijspp.2014-0329, PMID: 25710259

[ref13] Gordon D. Wightman S. Johnstone J. Espejo-sanchez C. Beckford C. Boal M. (2017). Physiological and training characteristics of recreational marathon runners. J. Sports Med. 8, 231–241. 10.2147/OAJSM.S141657 PMC5703178 29200895

[ref14] Govus A. D. Coutts A. Duffield R. Murray A. Fullagar H. (2017). Relationship between pretraining subjective wellness measures, player load, and rating-of-perceived-exertion training load in American college football. Int. J. Sports Physiol. Perform. 13, 95–101. 10.1123/ijspp.2016-0714 28488913

[ref15] Haddad M. Stylianides G. Djaoui L. Dellal A. Chamari K. (2017). Session-RPE method for training load monitoring: validity, ecological usefulness, and influencing factors. Front. Neurosci. 11:612. 10.3389/fnins.2017.00612 29163016 PMC5673663

[ref16] Halson S. L. (2014a). Monitoring training load to understand fatigue in athletes. Sports Med. 44, 139–147. 10.1007/s40279-014-0253-z PMC4213373 25200666

[ref17] Halson S. L. (2014b). Sleep in elite athletes and nutritional interventions to enhance sleep. Sports Med. 44(Suppl. 1), S13–S23. 10.1007/s40279-014-0147-0 24791913 PMC4008810

[ref18] Hernández-Cruz G. López-Walle J. M. Quezada-Chacón J. T. Jaenes Sánchez J. C. Rangel-Colmenero B. R. Reynoso-Sánchez L. F. (2017). Impact of the internal training load over recovery-stress balance in endurance runners. J. Sport Psychol. 26, 57–62.

[ref19] Hooper S. L. Mackinnon L. T. (1995). Monitoring overtraining in athletes. Sports Med. 20, 321–327. 10.2165/00007256-199520050-00003, PMID: 8571005

[ref20] Hopkins W. G. Marshall S. W. Batterham A. M. Hanin J. (2009). Progressive statistics for studies in sports medicine and exercise science. Med. Sci. Sports Exerc. 41, 3–13. 10.1249/MSS.0b013e31818cb278, PMID: 19092709

[ref21] Impellizzeri F. M. Rampinini E. Marcora S. M. (2005). Physiological assessment of aerobic training in soccer. J. Sports Sci. 23, 583–592. 10.1080/02640410400021278 16195007

[ref22] Kumar P. (2015). Effect of fartlek training for developing endurance ability among athletes. Int. J. Phys. Educ. Sport. Heal. 2, 291–293.

[ref23] Malone J. J. Di Michele R. Morgans R. Burgess D. Morton J. P. Drust B. (2015). Seasonal training-load quantification in elite English premier league soccer players. Int. J. Sports Physiol. Perform. 10, 489–497. 10.1123/ijspp.2014-0352 25393111

[ref24] Malone S. Hughes B. Roe M. Collins K. Buchheit M. (2017). Monitoring player fitness, fatigue status and running performance during an in-season training camp in elite Gaelic football. Sci. Med. Football 1, 229–236. 10.1080/24733938.2017.1361040

[ref25] Manzi V. D’Ottavio S. Impellizzeri F. M. Chaouachi A. Chamari K. Castagna C. (2010). Profile of weekly training load in elite male professional basketball players. J. Strength Cond. Res. 24, 1399–1406. 10.1519/jsc.0b013e3181d7552a 20386474

[ref26] Phibbs P. J. Jones B. Roe G. Read D. Darrall-Jones J. Weakley J. (2018). The organised chaos of English adolescent rugby union: Influence of weekly match frequency on the variability of match and training loads. Eur. J. Sport Sci. 18, 341–348. 10.1080/17461391.2017.1418026 29303682

[ref27] Roos L. Taube W. Beeler N. Wyss T. (2017). Validity of sports watches when estimating energy expenditure during running. BMC Sports Sci. Med. Rehabil. 9, 1–8. 10.1186/s13102-017-0089-6 29296281 PMC5738849

[ref28] Roos L. Taube W. Brandt M. Heyer L. Wyss T. (2013). Monitoring of daily training load and training load responses in endurance sports: what do coaches want? Schweiz. Z. Med. Traumatol. 61, 30–36.

[ref29] Rowlands D. S. Pearce E. Aboud A. Gillen J. B. Gibala M. J. Donato S.. (2012). Oxidative stress, inflammation, and muscle soreness in an 894-km relay trail run. Eur. J. Appl. Physiol. 112, 1839–1848. 10.1007/s00421-011-2163-1, PMID: 21922261

[ref30] Saugy J. Place N. Millet G. Y. Degache F. Schena F. Millet G. P. (2013). Alterations of neuromuscular function after the World’s most challenging mountain ultra-marathon. PLoS One 8:e65596. 10.1371/journal.pone.0065596, PMID: 23840345 PMC3694082

[ref31] Stellingwerff T. (2012). Case study: nutrition and training periodization in three elite marathon runners. Int. J. Sport Nutr. Exerc. Metab. 22, 392–400. 10.1123/ijsnem.22.5.392, PMID: 23011657

[ref32] Twist C. Highton J. (2013). Monitoring fatigue and recovery in Rugby league players. Int. J. Sports Physiol. Perform. 8, 467–474. 10.1123/ijspp.8.5.467 23319463

[ref33] Vernillo G. Savoldelli A. Skafidas S. Zignoli A. La Torre A. Pellegrini B. (2016). An extreme mountain ultra-marathon decreases the cost of uphill walking and running. Front. Physiol. 7:530. 10.3389/fphys.2016.00530 27877137 PMC5100553

